# Non-Negligible Effects of UV Irradiation on Transformation and Environmental Risks of Microplastics in the Water Environment

**DOI:** 10.3390/jox12010001

**Published:** 2021-12-21

**Authors:** Fangyuan Cheng, Tingting Zhang, Yue Liu, Yanan Zhang, Jiao Qu

**Affiliations:** State Environmental Protection Key Laboratory of Wetland Ecology and Vegetation Restoration, School of Environment, Northeast Normal University, Changchun 130117, China; chengfy310@nenu.edu.cn (F.C.); zhangtt671@nenu.edu.cn (T.Z.); liuy649@nenu.edu.cn (Y.L.)

**Keywords:** MPs, photoaging, ultraviolet light, influence factors

## Abstract

Microplastics (MPs) are ubiquitous in environmental media, and their harmful effects on MPs on the ecosystem have attracted more and more attention. Once released into the environment, MPs can trigger oxidative degradation through ultraviolet (UV) to cause photoaging. Photoaging significantly affects the properties of MPs, which leads to changing their environmental behaviors and increasing environmental risks. In this review, the generation of MPs under UV irradiation and the influence of environmental factors on the photoaging of MPs were discussed. Photoaging of MPs is an important process affecting the migration, transformation and interaction of pollutants in water and soil. In order to fully predict the fate and environmental interaction of MPs, more researches are needed in the future to explore the photoaging behavior of different types of MPs under natural environmental conditions.

## 1. Introduction

As one of the most important materials in the 21st century, plastics have an impact that cannot be underestimated on our society and daily life [1,2,3]. Since the 1950s, a variety of plastic products have continued to be released, and the output of plastics has increased exponentially. In 2015, the global output of plastic products reached 322 million tons, and the total output is expected to reach about 600 million tons by 2025 [4]. Due to the large-scale production and poor management of plastics in the past few decades, most plastic products are discarded after use, and only 6–26% are recycled and landfilled [5,6]. Large amounts of plastic waste migrate to the ocean with surface runoff and float, deposit or accumulate in coastal waters such as estuaries and bays [2,3,7]. It is estimated that 6–12 million tons of plastic enter the ocean every year, and more than 250 million tons of plastic will accumulate in the ocean by 2025 [8,9]. Plastic products have become the main component of marine garbage, accounting for about 60–80% of its total, and even as high as 95% in some areas, which causes serious pollution to the marine environment [2,3,10].

According to statistics, the most commonly used plastics are polystyrene (PS), poly(ethylene terephthalate) (PET), polyurethane (PUR), poly(vinyl chloride) (PVC), polyethylene (PE) and polypropylene (PP), which accounted for about 81% of European plastic demand in 2016 [11,12,13,14,15]. MPs (particle size <5 mm) have attracted widespread attention as a new type of pollutant in recent years [16,17,18]. A part of the MPs in the environment comes from the direct discharge of sewage treatment plants and urban pipe networks [16,19], such as fibers in clothing and plastic microbeads in care products, and the other part mainly comes from the weathering and degradation of large pieces of plastics [17,20]. The plastic fragments in the environment are separated and broken under the effects of ultraviolet (UV) radiation, physical wear and biological habitat and gradually form MPs [16,18,21,22,23]. The process of plastic degradation progresses with time and eventually leads to the formation of nanoplastic (NPs, particle size <1 μm) [24,25].

With the investigation of the distribution of MPs in the environment and the exploration of their biological toxicity, the pollution of MPs in environmental media has become one of the issues that researchers are highly concerned about. The distribution of MPs has spread to every corner of the earth; humans have found traces of it in the coastal estuaries, oceans, equatorial waters and north and south poles [16,17,18,19,20,21,22,23,24,25,26,27,28].

Statistics showed that the abundance of MPs in the coastal waters of China is 0.13–545 pieces/m^3^ and that in estuary sediments is 20–7900 pieces/kg [29]. In the seawater at a depth of 2673–10,908 m and the sediment at a depth of 5108–10,908 m in the Mariana Trench, the contents of MPs are 2.06–13.51 pieces/L and 200–2200 pieces/kg, respectively. It is very likely to pose a potential threat to the fragile and unique ecosystem of the abyss due to the widespread and persistent distribution of MPs in the trench [27]. At the same time, MPs pollution in the freshwater environment cannot be ignored. It is estimated that 80% of MPs in the ocean come from land [2,9], and rivers are one of the main ways for plastics to reach the ocean [19,30].

MPs are considered potential sources of chemical contaminants due to the release of intermediates during the aging process of MPs. Plastics contain various chemical additives, including stabilizers, plasticizers, fillers and antioxidants, which are released into the environment during the process of plastic fragments forming MPs [8,25,31]. A large number of researches proved that as a good carrier for adsorbing organic pollutants, MPs could transfer persistent, bioaccumulative and toxic harmful substances to the food chain, seriously endangering the ecological environment and human health [17,25,32,33]. Therefore, it is of great significance to understand the transformation behavior of MPs in the environment.

MPs undergo aging in the environment, and the aging process mainly includes mechanical breakage, photo-degradation, thermal degradation and biodegradation. The combined influence of various environmental factors on the MPs’ aging process is complex, leading to unpredictable changes in the fate of MPs in the environment. UV light is considered to be the most critical factor in the aging process of polymers. The chemical oxidation induced by light is enhanced, showing a synergistic aging effect. Polymer photo-oxidation reaction generally includes four processes: chain initiation, chain growth, branch formation and chain termination. The photo-degradation of polymer is when unsaturated structure or additives absorb UV light to form free radicals and then interact with oxygen to generate more free radicals, which accelerates its aging. Under the combined action of UV light and oxygen, the aging speed of the polymer would be further accelerated and eventually lead to the collapse of the polymer chain [34]. Hence, the impact of UV light on the environmental behavior of MPs was discussed in this review to clarify the key role of UV-induced photoaging in affecting the environmental behavior of MPs. This review provides valuable information for evaluating the potential environmental transformation of MPs in the natural environment, with a view to providing a reference for the theoretical research, risk assessment and control technology of MPs pollution.

## 2. Production of MPs under UV Irradiation

Understanding the impact of UV on the fragmentation process and mechanism of plastics is of great significance for predicting the production of MPs in the environmental medium. Current research proved that the chemical oxidation of MPs could be enhanced by light, showing a synergistic aging effect. Polymer photo-oxidation reactions generally include chain initiation, chain growth, chain formation and chain termination. Under the combined action of UV irradiation and oxygen, the aging rate of the polymer will be further accelerated. The photo-degradation of polymer is initially due to the unsaturated structure or additives in the polymer absorbing UV light, forming free radicals, which then interact with oxygen to produce more free radicals, thus accelerating its aging and eventually causing the polymer chain to collapse [34].

### 2.1. UV Irradiation Induced Cracking of Plastics

UV irradiation can change the surface roughness, morphology and chemical properties of plastics, accelerate the weathering process of plastics seriously and eventually lead to the formation of MPs [35]. Song et al. [36] studied the comprehensive effects of UV exposure time and mechanical abrasion on MPs breaking and found that the combined effect of UV exposure for 6 months and mechanical abrasion for 2 months can produce 12,152 ± 3276 plastic particles, and a part of particles broke into undetectable sub-micron particles at the same time. After placing PP, PE and PET in water and irradiating with UV-254, more and more particles with smaller diameters were observed [37]. The average particle size of the original PS was reduced from 55.9 μm to 38.6 μm under 60 days of UV irradiation, resulting in 7.03 ± 0.37% of NPs (~75 nm), which led to surface oxidation and persistence of PS particles [38]. 

The surface morphology of microfibers changed after UV light and cracked to produce smaller particles in the marine environment [39]. UV irradiation and mechanical wear promote the fracture and photo-oxidation of polycarbonate MPs (PC-MPs) [40]. The above research results all proved that photo-degradation is an important source of MPs in the ecosystem, and photo-chemically induced brittleness can lead to the formation and release of small particles from plastics [37,41]. Recent studies on the photoaging process of MPs were summarized in Table 1, indicating that most experiments were carried out under laboratory conditions.

### 2.2. Common Photoaging Forms of MPs

During the UV degradation process, the oxidation of plastic particles, the generation of surface cracks and the formation of fragments are common photoaging forms [42,48,49,50]. PS, PF, PE and PVC undergo photo-oxidation under simulated sunlight, accompanied by the rupture of C–C and C–H bonds [43]. At the same time, UV light is an important factor in the dechlorination of PVC [51]. It was demonstrated that the polymers’ surface of low-density for PE, PP and PS were broken during 60 days of UV radiation, and new carbonyl groups, vinyl groups and hydroxyl groups were formed [44]. Xiong et al. used ultraviolet radiation to simulate the aging process of PS under natural conditions and found that the carbonyl index increased significantly from 0.183 to 0.387 after 120 h [52].

Under UV irradiation, new carbonyl groups, vinyl groups, hydroxy peroxides and obvious local micro-cracks are generated on the plastic surface, which reduces the performance of the plastic and increases its brittleness [45,53]. The aging film obtained under laboratory conditions was similar to the aging film obtained under natural conditions. UV light played a vital role in the aging of the film. With the extension of the natural aging time, cracks and oxygen-containing functional groups continue to appear on the surface of the film, which leads to the decrease in its mechanical strength and the formation of MPs [46].

The COVID-19 pandemic has promoted explosive growth in the use of masks. According to research, the chain structure and chemical composition of PP in masks change during the UV aging process, and a mask could release more than 1.5 million particles into the water environment [54]. The chemical impurities such as carbonyl groups, unsaturated carbonyl groups, carboxylic acids and hydrogen peroxide derivatives in polyolefins make them prone to photo-degradation. Meanwhile, the length of the side chain affects the stability of different polyolefins to photo-oxidation and degradability. As a result of the above reasons, polyolefins degrade and generate MPs under long-term exposure to UV light [55]. UV aging significantly affected the physical and chemical properties of PE, which included an increase in hydrophilicity and crystallinity, reduction in average particle size and introduction of oxygen-containing functional groups [47]. Photoaging is mainly dominated by the generation of reactive oxygen species (ROS) in the environmental media [40]. The molecular weight decrease in plastics caused by photo-induced oxidation and the formation of oxygen-containing functional groups such as carboxyl, hydroxyl and carbonyl groups make the materials more brittle and increase the possibility of bio-degradation and mechanical degradation [34,51].

## 3. Factors Affecting Photoaging of MPs in the Environment

The various photochemically reactive components, such as Cl^−^, Br^−^, NO_3_^−^, CO_3_^2−^, SO_4_^2−^ and natural organic matter, existing in the environment would affect the photoaging reaction of MPs in the environment.

### 3.1. The Effect of Organic Matter on Photoaging of MPs

Previous researches showed that sulfides that are ubiquitous in the environment could accelerate the oxidation of MPs during UV irradiation, which induced physical damage (embrittlement and cracking) and chemical conversion (increased O/C ratio and formation of C–S) of MPs. The spontaneous oxidation of sulfides leads to the formation of hydroxyl free radical (•OH), which is an oxidizing agent that attacks the carbon atoms in the polymer chain, causing the surface oxidation and chain scission of polymers [56]. Therefore, the presence of sulfide under irradiation would promote the generation of free radicals in the system, which thus promote the surface oxidation of MPs. Dissolved organic matter (DOM) in the aquatic environment and soil organic matter (SOM) in the terrestrial environment play a significant role in the photo-degradation and bio-degradation of MPs [57]. DOM and SOM can be used as photosensitizers due to the rich content of chromophores (such as aromatic rings and carboxyl groups). Considering that the ROS was generated by DOM photosensitization to promote the oxidation of the polymer, the difference of DOM content in the waters of the Yangtze River and Taihu Lake may be one of the important factors leading to the difference in the photoaging rate of PS or PE [58]. The light-shielding effect of biofilms in the environment would inhibit the photo-degradation of MPs [59]. The presence of low molecular weight organic acid (LMWOA) and LMWOA-Fe(III) complexes could promote the photoaging reaction of PVC-MP [60]. •OH was produced by photolysis of LMWOA, or its iron complex played a leading role in the degradation of PVC-MP. The C–Cl bond fracture occurred on the surface of PVC-MP, forming oxygen-containing functional groups such as carbonyl groups, especially in the presence of LMWOA and LMWOA-Fe(III) [60]. The effect of low-molecular-weight organic compounds such as benzophenone (BPh), anthraquinone (AQ) and benzoyl peroxide (BPo) on the degradation processes of polystyrene was studied, which could accelerate and increase the photo-degradation and photo-oxidation of polystyrene [61].

### 3.2. Effect of Inorganic Substances on Photoaging of MPs

The presence of inorganic substances in the environment could also affect the photoaging behavior of MPs. For example, iron oxide inhibited the photoaging of plastic by reducing its light absorption efficiency. Iron red pigment reduced the photoaging rate of PP-MPs significantly, which mainly depended on the reaction of Fe(III) with organic acids and reactive oxygen species derived from MPs under irradiation conditions [62,63,64]. However, Neubauer et al. discovered that iron-red pigment accelerated the photo-oxidation of PE plastics under the treatment of UV and rainwater and speculated that iron as an electron acceptor might mediate the generation of ROS and the formation of hydrogen peroxide [65]. These researches reveal the dual influence of iron oxide pigments on the photoaging behavior of MPs and highlight the pivotal role of inorganic pigments in the environmental fate and risk of MPs.

On account of the variability in water composition, the photoaging processes and mechanisms of MPs in different water environments are complex. Salinity ions affect the photo-degradation rate of MPs in aquatic ecosystems by influencing UV penetration depth and free radical reaction, which also leads to the otherness of the photoaging mechanism of MPs in the freshwater system and seawater system. The increase in salt concentration led to the high refractive index of water and salt crystals that formed on the surface of MPs; thereby, the inhibition of the MPs photo-degradation rate was caused by the reduction in the light absorption efficiency [42,66]. At the same time, Cl^−^ could also effectively sequestered •OH radicals, which may weaken the promotional effect of •OH on the photoaging of MPs [67]. The research of Ranjan et al. proved that PP in the terrestrial environment became fragmented within one month and was difficult to recycle, but the PP in the marine environment was less weathered and could be recycled [68]. The photoaging rates of PP-MPs in ultrapure water, estuary water and seawater were various, of which the aging rates of PP-MPs in estuary water and seawater were significantly lower than that in ultrapure water. In addition, the carbonyl index decreased with the increase in the concentration of Cl^−^. This is due to the reaction of Cl_2_^•−^ generated in seawater under irradiation conditions with HO_2_• to prevent the formation of O_2_^•−^, thereby inhibiting the photoaging process of PP-MPs [69]. Nevertheless, the common trace metal ions in natural water (such as iron and manganese) are displayed as catalysts to promote the photo-oxidation and chain scission of plastics under irradiation [60,70].

## 4. Environmental Risks of MPs after Photoaging

### 4.1. Photoaging Affects Migration of MPs in Environmental Media

Photoaging significantly affects the physical and chemical properties of MPs, and further influences their transportation and fate in the environment. Therefore, how the aging of MP under UV radiation further exacerbated its negative impact on the environment was summarized in the review. The aging process caused by UV light could alter the surface charge density and even the Hamaker constant of plastics, thus affecting their aggregation behavior in the environment [71]. Previous studies demonstrated that NPs could be transferred from the marine environment to the atmosphere by the rupture of bubbles on the sea surface and that the mobility of UV-aged PP-NPs was higher than that of incipient PP-NPs [72]. The photoaged MPs have a stronger migration ability and a larger cumulative flux ratio in the soil profile. Ren et al. found that the migration rate of photoaged MPs (34.9–89.2%) was higher than that of original MPs (30.5%) in sandy and clay loam [45]. Photoaging improved the mobility of spherical polystyrene NPs (PS-NPs, 487.3 ± 18.3 nm) in saturated loam sand and the adsorption capacity of pollutants, which is mainly attributed to the surface oxidation of NPs. These research results provide new insights into the fate and transportation of MPs in natural media and their potential risks to the groundwater.

### 4.2. Interaction of Photoaging MPs and Pollutants

Previous studies testified that the interaction between MPs and organic pollutants is influenced by hydrophobic forces [51,73,74,75,76]. During the photoaging process, the rise of oxygen-containing functional groups on the surface of MPs leads to an increase in surface electronegativity and hydrophilicity, which obviously impacts the transportability of MPs toward pollutants [77]. Thus, it is significant to realize the adsorption behavior and mechanism of photoaging MPs and organic pollutants for evaluating the ecological risks of MPs in the environment.

The photoaging process could change the surface properties of MPs and affect the adsorption capacity of hydrophilic and hydrophobic organic pollutants [73,78,79]. The increase in oxygen-containing functional groups on the surface of photoaged MPs may generate the formation of hydrogen bonds between the surface of MPs and surrounding water molecules. It is difficult for the hydrophobic organic pollutants to replace the water molecules adsorbed by MPs, thus reducing the adsorption sites on the surface of MPs [80,81]. Due to the large number of oxygen-containing functional groups generated on the PS surface after UV irradiation, the adsorption capacity of the photoaged PS surface to 2,2′, 4,4′-Tetrabromodi phenyl ether (BDE-47) was lower compared with that of the original PS [80]. However, the adsorption capacity of PE particles irradiated by UV light to polycyclic aromatic hydrocarbons (PAHs) with high hydrophobicity was slightly higher than that of the initial PE as the π–π effect between PAHs and PE was enhanced by the introduction of oxygen groups on the surface of the original PE during UV irradiation process [82]. UV aging treatment also improved the adsorption of phenanthrene (Phe) by polyurethane (PT) and urea-formaldehyde resin (UF) [83]. Consequently, the adsorption behavior of hydrophobic organic compounds on aging MPs is not only related to the chemical properties of the organic compounds but also closely related to the types physical and chemical properties of MPs.

The photoaged MPs can also enhance the adsorption capacity for hydrophilic organics. Among them, PS and PVC after photoaging by UV irradiation showed higher adsorption of ciprofloxacin (CIP) than unaged MPs, and the main mechanism of adsorption between photoaged MPs and CIP was the physical interaction (electrostatic interaction and intermolecular hydrogen bond interaction) [51,60]. The specific surface area and hydrophilicity of PLA increased after ultraviolet ray aging, which contributed to an increase in the adsorption capacity of antibiotics (CIP and tetracycline) [84]. After photoaging treatment of PS, the equilibrium adsorption capacity of CIP and bisphenol A (BPA) increased from 0.15 and 4.07 mg/g to 4.92 and 8.71 mg/g, respectively [49]. Besides, the photoaged MPs would also have a certain impact on the conversion of organic matter. Wang et al. demonstrated that the presence of photoaged PVC-MPs accelerated the hydrolysis of cefazolin (CFZ) but exhibited a slight effect on the degradation of cephalexin (CFX). For this reason, while the increase in adsorption capacity for antibiotics, the aged PVC-MPS could be used as a catalyst to participate in the degradation of antibiotics [85]. In addition, the impact of photoaged MPs that adsorb hydrophilic organics on organisms needs to be further explored [51,60].

MPs are good carriers of heavy metals in the environment, and photoaging can also affect the adsorption performance of MPs to heavy metals. The adsorption capacity of MPs for heavy metals after photoaging is higher than that of original MPs, which was speculated to be related to the increase in specific surface area of aging MPs and the generation of oxygen-containing functional groups [33,86]. With the extension of the irradiation time, the adsorption capacity of PS-MPs toward Cu^2+^ and Zn^2+^ increased, and the adsorption capacity and adsorption strength of PP-MPs and tire wear particles for Pb^2+^ and Cd^2+^ rose. The results of the above researches proved that the correlation between the aging degree of MPs and the adsorption capacity of heavy metals was existed [33,84,86,87].

### 4.3. Additives in MPs Released into the Environment

Additives and life-extension compounds mainly function through the following five ways: (1) light shielding, (2) ultraviolet absorption, (3) excited state quenching, (4) peroxide decomposition and (5) free radical scavenging [61]. Photochemical weathering causes degradation of MPs and releases degradation by-products such as chemical additives into the environment. Three types of PVC-MPs with different particle diameters could release organotin compounds (OTCs) under UV and visible light irradiation. The presence of DOM in the environment facilitated the release of OTCs in MPs, which is due to the excited triplet state of DOM (^3^DOM*) generated by DOM under irradiation conditions that promoted the photoaging reaction of PVC [67].

UV light irradiation accelerated the leaching of dissolved organic matter in MPs (MP-DOM), and the trihalomethane (THM) produced by the chlorination reaction of MP-DOM was harmful to the environment [46]. MP-DOM could be adsorbed on the mineral surface and affect its surface properties and reaction activity with pollutants, which was similar to natural organic matter [46]. The concentration of heavy metals in the leachate of PVC exposed to UV-C radiation was higher than that of unweathered PVC [88]. The increase in the absorbance of the C–Cl bond in PVC after degradation indicated the occurrence of Cl migration, which brought about the rechlorination of the intermediate chlorophenol produced [89]. A series of molecular degradation products (sulfur compounds, terephthalic acid, 4-acetylbenzoic acid, etc.) and additives (bisphenol A, butylbenzene sulfonamide, triphenyl phosphate, diphenylmethyl, etc.) were detected in the seawater leachate of microfibers during the photoaging process [40].

### 4.4. Toxic Effects of Photoaging MPs on Organisms

Toxicological studies on MPs proved that MPs have certain toxic effects on organisms. Nevertheless, MPs exposed to ultraviolet radiation for a long time have a stronger toxic effect on organisms. Compared with raw PS-MPs, long-term exposure to photoaged PS-MPs solution at low concentration (1 μg/L) resulted in stronger neurotoxicity to nematodes, which is due to the neurotoxicity of aged PS-MPs by affecting the neurotransmission of dopamine, glutamate and serotonin [90]. The photoaged PP and PS further reduced the enzyme activity of the microorganisms in the soil, so the photoaged MPs would more significantly affect the environmental function of the soil [91].

The organic matter (benzaldehyde, benzoic acid, etc.) leached from PS after UV and UV/H_2_O_2_ treatment increased its toxicity to bacteria [92]. The aged PVC after UV irradiation showed a significant inhibitory effect on the growth rate of freshwater algae compared with the incunabular PVC [93]. Compared with the pristine PS, photoaged PS displayed a higher effect on the growth inhibition and lipidation damage of grouper (*Epinephelus moara*) [38]. These findings illustrated the potential hazards of photoaging MPs to organisms and ecosystems, which facilitated insight into the potential environmental risks of photoaged MPs.

## 5. Research Prospects

To better understand the ecological and environmental risks of photoaging of MPs, more attention should be paid to the following aspects in future studies:

It is encouraged to collect MPs samples from the environment for surface chemical analysis, revealing the changes in the adsorption behavior of MPs with different degrees of photoaging toward different types of organics. The impact of NPs produced by MPs in the photoaging process on the groundwater environment and soil ecosystem should also be paid more attention to in the future.

The current researches on the photoaging process of MPs are mostly carried out in artificial laboratory simulation or under the natural control conditions of a separate system, which is different from the actual natural environment under the combined action of multiple environmental factors. Hence, the researches on the photoaging process of MPs in natural environments need to be strengthened urgently. It is recommended to conduct long-term simulation experiments of MPs under outdoor environmental conditions. In addition, long-term experiments are needed to reveal further the biophysical and chemical interactions between photoaged MPs and biological communities in the environment.

The photoaging mechanism of MPs is understood to a certain extent, but it is more at the level of qualitative description. There is still a lack of quantitative model verification for the aging relationship between MPs and various environmental factors. While the aging mechanism of MPs is being further explored, a parameterized model is urgently needed to be established in order to quantitatively analyze the contribution rate of various environmental factors to the photoaging of MPs.

## Figures and Tables

**Table 1:** Photoaging process of MPs under different environmental conditions.

MPs	Experimental Conditions				Conclusion	Reference
	Size	Time	Matrix	Method		
PE, PP, PS	PE: 26 ± 0.8 mm PP: 19 ± 0.9 mm PS: 22 ± 2.2 mm	UV exposure: 12 months Mechanical abrasion (MA): 2 months.	simulate a beach environment	Metal halide lamp (UV-A: 11.01 W/m^2^; UV-B: 0.12 W/m^2^; UV-C: 0.04 W/m^2^) and MA	The surface of theMPs pellets yellowed, became fragile and brittle and showed cracks; particles had fragmented.	[36]
PP, PE, PET	~0.025 mm	72 h	ocean water	254 nm UV light	Smaller particles were produced.	[37]
PS	20–100 μm	60 d	natural seawater	UVA/B (113 ± 45 W/m^2^)	The particle size of PS decreased, resulting in the formation of nanoparticles (~75 nm), surface oxidation and formation of persistent free radicals can be observed.	[38]
PET, PA, wool yarns	~2 mm	56 d	Seawater	Xenon lamp (1500 W, 65 W/m^2^)	The surface was broken, micron-size particles were formed and additives of degradation products were detected in the leachate.	[39]
PC	12.58 μm	640 h	water	500 W mercury lamp (11.0 mW/cm^2^ at 365 nm)	Light and mechanical wear promote the fracture and photo-oxidation of PC-mps, and the continuous degradation of the polymer is accompanied by a sharp decrease in molecular weight.	[40]
PS	—	5 d	water	Mercury lamp	The surface of PS particles became rough, embrittlement and cracking.	[41]
PE, PP, PS	—	3 months	air, seawater, ultrapure water	UVA-340	The surface of plastic particles was oxidized, cracks and fragments appeared.	[42]
PS, PF, PE, PVC	PS: 95.0 ± 5.0 μm PF: 66.5 ± 6.2 μm PE: 110.0 ± 10.0 μm PVC: 115.0 ± 5.0 μm	15 d	ambient atmosphere	Xenon lamp (2.85 mW/cm^2^)	Chemical chain scission, several environmentally persistent free radicals (EPFRs) were detected.	[43]
PE, PP, PS	350 ± 30 μm	60 d	ambient atmosphere	UV irradiation (500 W/m^2^)	Low molecular weights molecules were formed, macromolecular chain scission provoked by oxidation and formation of new functional groups, such as vinyl, carbonyl, hydroxyl/hydroxyperoxide segments.	[44]
PS	1 μm	8 d	water	15 W UVA-340 lamp	The surfaces of the MPs were becoming rough and uneven, the average particle size of UV-PS MPs decreased and the oxygen-containing functional groups were produced.	[45]
PE	0.015 mm and 0.006 mm	15 d	ambient atmosphere	100 W mercury lamp	Cracks and oxygen-containing functional groups occurred on the surface of the film.	[46]
PE	6.00 to 8.50 µm	7 d	Seawater	UV-254	The hydrophilicity and crystallinity of PE particles increased, the average particle size decreased, and oxygen-containing functional groups were formed on the surface of PE.	[47]

Note: MPs: Microplastics; PE: polyethylene; PP: polypropylene; PS: polystyrene; PF: phenol-formaldehyde resin; PVC: poly(vinyl chloride); PET: poly(ethylene terephthalate); PA: polyamide.
